# Extent of male involvement and associated factors in antenatal care service utilization in Bench Sheko zone, Southwest Ethiopia: A community-based cross-sectional study

**DOI:** 10.3389/fgwh.2022.938027

**Published:** 2022-12-02

**Authors:** Mengistu Mekonen, Nigusie Shifera, Biruk Bogale, Ashenafi Assefa

**Affiliations:** ^1^ Department of Public Health, School of Public Health, College of Medicine and Health Science, Mizan Tepi University, Mizan Aman, Ethiopia; ^2^ Department of Nursing, College of Medicine and Health Science, Mizan Tepi University, Mizan Aman, Ethiopia

**Keywords:** male involvement, antenatal care, Bench Sheko, Ethiopia, southwest Ethiopia

## Abstract

**Background:**

In many low- and middle-income countries (LMIC), men are the key decision-makers and chief providers, often determining women's access to economic resources and maternal health services. Despite the important role of men in maternal healthcare, the involvement of male partners in maternal and child health in LMIC, including Ethiopia, is low.

**Objectives:**

This study aims to assess the extent of male involvement and associated factors in antenatal care (ANC) service utilization in the Bench Sheko zone, Southwest, Ethiopia.

**Methods:**

A community-based, cross-sectional study was conducted in the Bench Sheko zone from February to May 2021. A multistage sampling technique was employed to select study participants. Variables with *p*-values <0.25 in binary logistic regression were selected as candidates for multiple logistic regression to determine independent factors associated with male involvement adjusting for sociodemographic, health service, personal, environmental, and knowledge-related factors.

**Results:**

A total of 816 men took part in the study, with a 98.2% response rate. The prevalence of male involvement in ANC utilization was 38.2%. In multivariate analyses, residence (urban), age (25–34), education (diploma and above), income (≥2,500 Ethiopian Birr, ETB), and high knowledge of the advantages of ANC were shown to be positively associated with male involvement in ANC utilization.

**Conclusion:**

Male involvement in ANC utilization was low. Considering the importance of male involvement in maternal healthcare, we advocate for policies and strategies that can improve knowledge of the advantages of ANC among men and can enhance their engagement in maternal care. Special attention should be given to younger partners and those partners who live in rural areas.

## Introduction

Male involvement in antenatal care (ANC) utilization can encompass many things including discussing maternal health issues with one's spouse/partner, making a joint decision as a couple about the spacing of a pregnancy, utilization of maternal services, and accompanying one's partner to seek ANC in a health facility (1). In the late 1990s, there was increased recognition of the importance of including men in maternal and newborn health (MNH) programs. Research indicates several mechanisms by which male involvement in MNH can support improved maternal and child health outcomes (2, 3). For example, engaging men in maternal and newborn health can increase care-seeking, improve home care practices, and support more equitable couple communication and decision-making for maternal and newborn health (4).

Male involvement enables men to support their spouses to utilize obstetric services and for the couple to adequately prepare for birth complications. This leads to a reduction in all three phases of obstetric delay: delay in the decision to seek care, delay in reaching care, and delay in receiving care (5). Male partners can play a crucial role in the first and second phases of delay in low- and middle-income countries (LMIC), and thereby positively impact birth outcomes (6,7).

In patriarchal communities in LMIC, men are the key decision-makers and chief providers, often determining women's access to economic resources (5). Women initiate ANC, but men often make decisions about care utilization (8). Male involvement in reproductive health has been promoted as a promising strategy for improving maternal and child health (MCH) as male involvement can play a vital role in ensuring safe pregnancy, delivery, and moral support to women (9, 10). However, more limited attention has been placed on male partners' involvement in maternity care in LMIC (5, 9). A study conducted in the North Gondar zone, Northwest Ethiopia, found that men's level of knowledge about obstetric danger signs, being married, completing college education, escorting their wife to antenatal care, and urban residence were predictors of male involvement in the maternal healthcare system (11).

The tendency to view maternal health as a woman's issue has contributed to a narrow focus that targets mostly women in intervention efforts. Most MCH programs seek to address the health needs of women and children by engaging and educating pregnant women and mothers in care-seeking practices for themselves and their children. This has contributed to men being sidelined as far as reproductive health and MCH matters are concerned (6, 7, 12).

In Ethiopia, the levels of maternal morbidity and mortality are among the highest in the world. According to estimates by World Health Organization (WHO) and UNICEF, the maternal mortality rate (MMR) in 2017 was 401 per 100,000 live births (13), which is more than 40 and 60 times higher than that of Europe and Australia, respectively (10). According to the United Nations' new Sustainable Development Goals (SDGs), all countries should reduce their MMR by at least two-thirds. The average global target is an MMR of less than 70/100,000 live births by 2030. The supplementary national target is that no country should have an MMR greater than 140/100,000 live births (10).

Despite the important role of men in maternal health, the involvement of male partners in MCH in LMIC, including Ethiopia, is low. Only 23.1% male partners have physically entered the ANC room with their wives in Addis Ababa (14). Another study from Gondar reported that only 40.1% husbands were involved in HIV counseling and testing during their wife's pregnancy (15). Available studies have explored the perspectives of women, but often not men. Furthermore, previous studies were not comprehensively assessing the role of males in the ANC of their partners. The current study seeks to assess male involvement in ANC and associated factors in the Bench Sheko zone, Southwest Ethiopia.

## Methods

### Study setting and population

The study was conducted in the Bench Sheko zone, which is one of the zones of the Ethiopian Southwest region. Mizan Aman is an administrative city, which is located 585 km southwest of the capital, Addis Ababa. The Bench Sheko zone is divided into six woredas and two city administrations namely Semen Bench, Debub Bench, Shay Bench, Gidi Bench, Sheko, Guraferda woredas, Mizan Aman city administration, and Siz city administration. According to the zonal statistics office, the total population of the Bench Sheko zone is 681,549. The Bench Sheko zone has 1 hospital, 26 health centers, and 128 health posts. This community-based cross-sectional study design was conducted from February to May 2021. Male partners of women (whether they were in a formal marriage or informal union) in their reproductive years (18–49) were the source population.

### Sample size calculation and sampling technique

The sample size was computed based on the single population proportion formula, and using the prevalence of male partner's involvement in promoting ANC utilization in Ambo town (59.9%) (16), Z-value of 1.96 at a 95% confidence level, a margin of error of 5%, and 10% nonresponse rate. As we follow multistage sampling, we use design effect two. So the final sample size was 816. Multistage sampling techniques were used to select study participants. Simple random and cluster sampling were used in a step-wise way. Out of six woredas and two city administrations, two woredas (Shay Bench and Guraferda woreda) and one city administration (Mizan Aman city administration) were selected by a simple random sampling technique. Mizan Aman city administration has five kebeles, and among these kebeles, Addis Ketema, Shesheka, and Hibret kebeles were selected with a lottery method. In Guraferda woreda, we select 10 kebeles out of 30 kebeles with simple random sampling and in Shay Bench woreda, 6 of 21 kebeles were selected in the same fashion. Finally, all partners of a woman who has a child whose age was less than 1 year old and lived in the selected kebele were included. A list of women who delivered within 1 year was traced with the help of health extension workers in each kebele.

### Measures

Male involvement in ANC utilization was defined as the involvement of the male partner in (1) discussions with health professionals on the spouse's delivery location, and (2) whether the husband accompanied the woman at least once during an ANC check-up. If the male partner reported that he had done both of these things, he was classified as “Involved in ANC.” If he missed one of these activities, he was categorized as “Not involved in ANC.”

Level of knowledge about the advantages of ANC: seven questions regarding the definition of ANC, number of visits, the advantage of ANC follow-up, and bad outcomes of not having ANC follow-up were asked. Those participants who scored more than the mean value were considered to have high knowledge about the advantages of ANC utilization. Those participants who scored less than the mean value were considered to have low knowledge about the advantages of ANC utilization.

Attitudes toward ANC utilization: five questions that assess male partners' attitudes toward their female partner's ANC utilization were asked. For example, we asked, “Do you think that ANC follow-up is important for women?” All the respondents with cumulative scores equal to or more than the mean were categorized as having a favorable attitude. Men who answered less than the mean score were considered to have an unfavorable attitude toward the utilization of ANC.

Waiting time: If a partner reported that he had to wait for more than 30 min without talking to a healthcare provider starting from when he arrived at the health facility, we categorized it as a long waiting time. It was assessed by self-report of the husband.

### Data collection and analysis

Data were collected using interviewer-administered structured questionnaires that addressed sociodemographic characteristics, personal and environmental factors, and male partners' involvement in the ANC utilization of their spouses/partners, service delivery experience, knowledge about the benefits of ANC, and attitudes of men toward ANC utilization by women. The questionnaire was designed in English, then translated into Amharic, and then back-translated to English by a third person to check for consistency. The interview took place within the households of respondents, and on average, it took about 30 min to complete the interview.

Data were entered into Epi data version 4.4 and exported to Statistical Package for Social Science (SPSS) version 23 for analysis. Data exploration was conducted to examine different characteristics of the data. After data cleaning, descriptive statistics such as frequencies, proportions, and percentages were used to assess the categorical variables, while measures of central tendency and dispersion were used to summarize continuous data.

Bivariate logistic regression was carried out to select candidate variables for multivariate logistic regression analysis with a *p*-value <0.25. Candidate variables were entered into a multivariable logistic regression model using a backward method to identify the statistically significant factors for male involvement by controlling possible confounders. The degree of association between dependent and independent variables was assessed using odds ratios and a *p*-value < 0.05.

### Variables

The dependent variable was the male partner's involvement in ANC utilization resulting in a child who is less than 1 year old. The independent variables were sociodemographic variables (age, marital status, educational level, ethnicity, religion, occupation, and wealth status), obstetric and related factors (previous history of ANC visit with his partner, parity, and family size), cultural factors, health service-related factors (cost, waiting time, distance from home to health facility, quality of service, and affordability and accessibility of services), knowledge, and attitudes about ANC.

### Data quality assurance

Data collectors were trained on how to collect and handle data. Questionnaires were pretested on 5% of the sample in the Keffa zone before formal data collection began. After data collection, each questionnaire was given a unique code by the principal investigators. Double entry of data was used to decrease errors and 5% of the entered data were rechecked by comparing the entered data with the actual questionnaire. Any errors identified at this time were corrected. The principal investigator reviewed the completed questionnaires at the end of data collection every day for completeness, consistency, and to take corrective measures.

### Ethical issues

Ethical clearance was obtained from Mizan Tepi University’s Ethical Review Committee. Written consent was obtained from the study subjects, and the right of the respondents to withdraw or not to participate was respected. The anonymity and confidentiality of the data provided were strictly maintained. Participants were assured that their participation was voluntary.

## Results

### Demographic factors

A total of 816 men took part in the study, with a 98.2% response rate. About two-thirds (65.5%) of the respondents were aged 25–34 years. The mean age of respondents was 29.3 (SD ± 3.68) years. Most of the respondents (70.7%) lived in rural areas, and 41.2% completed primary level education. Almost half (46.6%) of study participants were involved in private sector work and 38.6% of respondents were Protestants. The majority of respondents (77.8%) were married (Table 1).

**Table 1:** Sociodemographic characteristics of male partners of women within the reproductive age group (18–49 years) in the Bench Sheko zone, Southwest region, Ethiopia, 2021.

Variables	Categories	Frequency	Percentage
Age category (mean = 29.3 SD ± 3.68)	15–24	106	13.2
	25–34	525	65.5
	≥35	170	21.2
Residential place	Urban	235	29.3
	Rural	566	70.7
What is your educational status?	Illiterate	128	16.0
	Primary	330	41.2
	Secondary	100	12.5
	Tertiary	161	20.1
	Diploma and above	82	10.2
What is your occupation?	Private work	373	46.6
	Farmer	99	12.4
	Merchant	186	23.2
	Student	129	16.1
	Government employee	11	1.4
	Daily laborer	3	0.4
What is your current marital status?	Not married	118	14.7
	Married	623	77.8
	Divorced	37	4.6
	Widowed	23	2.9
What is your religion?	Orthodox	272	34.0
	Protestant	309	38.6
	Catholic	65	8.1
	Muslim	143	17.9
	other	12	1.5
What is your Ethnicity?	Bench	500	62.4
	Kaffa	184	23.0
	Amhara	27	3.4
	Sheko	69	8.6
	Other	20	2.5
Average monthly income (in ETB)	<2,500	450	56.2
	≥2,500	351	43.8

ETB, Ethiopian Birr.

### Personal and environmental factors

The majority of men (78.4%) said that they decided on health-seeking behaviors together with their female partners. More than half (55.8%) of respondents have nearby health institutions (within 5 km radius) in their kebele, whereas 67.9% of respondents took more than 30 min to reach a health center (Table 2).

**Table 2:** Personal and environmental factors of male partners of women in their reproductive years (18–49 years) in the Bench Sheko zone, Southwest region, Ethiopia, 2021.

Variables	Categories	Frequency	Percentage
Who was the decision-maker for ANC healthcare seeking?	Husband only	173	21.6
	Both	628	78.4
Is there any nearby Health institution in your kebele?	Yes	447	55.8
	No	354	44.2
How long does it take to go to the nearest health institution (one way)?	<30 min	257	32.1
	>30 min	544	67.9
Had you heard about Community-Based Health Insurance (CBHI)^a^?	Yes	627	78.3
	No	174	21.7
Had you ensured in Community-Based Health Insurance (CBHI)?	Yes	483	60.3
	No	318	39.7

^a^ Community-Based Health Insurance (CBHI) is a type of health insurance for individuals who pay a certain fee annually and receive healthcare services freely for a year.

### Reasons why men did not attend ANC with their female partners

**Figure 1:**
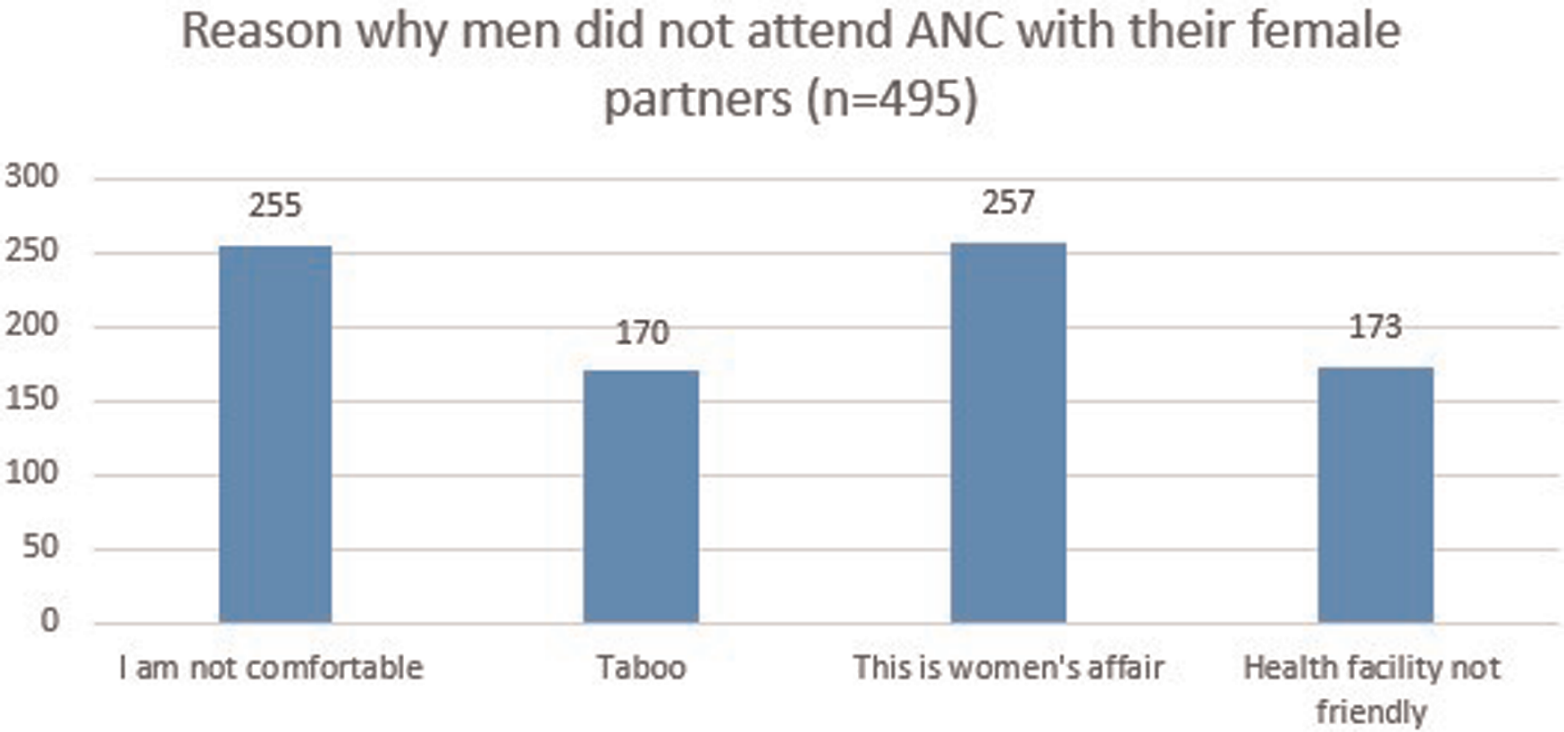
Reason why men did not attend ANC with their female partners in the Bench Sheko zone, Southwest region, Ethiopia, 2021. ANC, antenatal care.

About half (51.5%) of men did not attend ANC with their female partners because they were not comfortable going with their partners. A similar percentage of respondents (51.9%) thought that ANC is a women's affair (Figure 1).

*Participants could select more than one reason for not attending ANC with their female partner. Therefore, percentages do not sum to 100.*

### The extent of male involvement, service delivery, and knowledge-related factors

The prevalence of male involvement in antenatal care service utilization was 38.2%. About 44.6% of the respondents said they experienced a long wait time (characterized as waiting for more than 30 min without having contact with a healthcare provider) when they came with their female partner for ANC, and 42.8% of respondents said that healthcare professionals were not cooperative during a visit with their female partners (Table 3).

**Table 3:** Prevalence of male involvement, service delivery experience, knowledge, and attitudes of male partners of women with reproductive age group (15–49 years) in the Bench Sheko zone, Southwest region, Ethiopia, 2021.

Variables	Categories	Frequency	Percentage
Male involvement	Involved	306	38.2
	Not involved	495	62.8
Waiting time	Short^a^	444	55.4
	Long	357	44.6
Professional cooperation	Cooperative^b^	458	57.2
	Not cooperative	343	42.8
Knowledge	Low	221	27.6
	High	580	72.4
Attitude	Favorable	358	44.7
	Unfavorable	443	55.3

^a^ A partner waits for less than 30 min without talking to a healthcare provider starting from when he arrived at the health facility.

^b^ The partner is comfortable with the information, advice, care and support from healthcare professional. It is a self-report.

### Predictors of male involvement in ANC

Five variables, namely, residence (urban), age (25–34), education (diploma and above), income (≥2,500 Ethiopian Birr, ETB), and high knowledge of the advantages of ANC, were independently associated with male involvement in ANC in multivariable logistic regression models. Male partners who lived in urban settings had almost five times the odds of male involvement in ANC [adjusted odds ratio (AOR): 4.91, 95% CI: 3.39–7.11] compared to rural residents. The odds of male involvement were two times higher (AOR: 2.17, 95% CI: 1.20–3.92) among men aged 25–34 as compared to 15–24 years. Men who had a diploma or higher-level education had 3.6 times the odds (AOR: 3.66, 95% CI: 1.86–3.7.22) of male involvement in ANC as compared to those who were illiterate. Moreover, men with high knowledge of the advantages of ANC utilization as compared to those with low knowledge of ANC had three times the odds of male involvement in ANC (AOR: 3.12, 95% CI: 1.94–5.01), and men with an average monthly income ≥2,500 ETB had 2.8 times the odds of male involvement in ANC as compared to those with a monthly income below 2,500 ETB (AOR: 2 87, 95% CI: (1.98–4.15) (Table 4).

**Table 4:** Multivariate associations of male involvement in ANC in the Bench Sheko zone, southwest Ethiopia, 2021.

Variables	Male involvement		COR (95% CI)	AOR (95% CI)	*P*-value
	Yes	No			
	*N* (%)	*N* (%)			
Residence					
Urban	159 (52.0)	76 (15.4)	5.96 (4.28–8.31)	4.91 (3.39–7.11)	<0.001
Rural	147 (48.0)	419 (84.6)	1	1	
Age category					
15–24	22 (7.2)	84 (17.0)	1	1	1
25–34	212 (69.3)	313 (63.2)	2.58 (1.56–4.26)	2.17 (1.20–3.92)	0.01
≥35	72 (23.5)	98 (19.8)	2.80 (1.60–4.91)	1.63 (0.84–3.17)	0.149
Educational status					
Illiterate	34 (11.1)	94 (19.0)	1	1	1
Primary	118 (38.6)	212 (42.8)	1.54 (0.98–2.45)	0.71 (0.41–1.22)	0.21
Secondary	29 (9.5)	71 (14.3)	1.13 (0.63–2.02)	0.91 (0.46–1.79)	0.78
Tertiary	74 (24.2)	87 (17.6)	2.35 (1.42–3.87)	1.52 (0.84–2.76)	0.167
Diploma and above	51 (16.7)	31 (6.3)	4.55 (2.51–8.24)	3.66 (1.86–7.22)	<0.001
Occupation					
Private work	117 (38.2)	256 (51.7)	1	1	1
Farmer	44 (14.4)	55 (11.1)	1.75 (1.11–2.75)	1.19 (0.65–2.19)	0.556
Merchant	78 (25.5)	108 (21.8)	1.58 (1.10–2.27)	1.02 (0.63–1.64)	0.929
Student	64 (20.9)	65 (13.1)	2.15 (1.43–3.24)	1.89 (1.06–3.37)	0.029
Government employee	2 (0.7)	9 (1.8)	0.48 (0.10–2.28)	0.525 (0.09–2.94)	0.463
Daily laborer	1 (0.3)	2 (0.4)	1.09 (.098–12.18)	0.62 (0.02–16.92)	0.776
Ethnicity					
Bench	197 (64.4)	303 (61.2)	1	1	1
Kaffa	62 (20.3)	122 (24.6)	0.78 (0.55–1.11)	1.49 (0.92–2.42)	0.099
Amhara	9 (2.9)	18 (3.6)	0.77 (0.34–1.75)	0.56 (0.20–1.56)	0.271
Sheko	30 (9.8)	40 (8.1)	1.15 (0.695–1.91)	1.95 (1.01–3.75)	0.045
Others	8 (2.6)	12 (2.4)	1.02 (0.4–2.55)	1.74 (0.46–6.52)	0.408
				1.19 (0.37–3.83)	0.764
Religion					
Orthodox	97 (31.7)	175 (35.4)	1	1	1
Protestant	109 (35.6)	200 (40.4)	0.98 (.69–1.38)	3.35 (0.77–14.56)	0.106
Muslim	25 (8.2)	40 (8.1)	1.13 (.64–1.97)	2.72 (0.62–11.94)	0.184
Catholic	72 (23.5)	71 (14.3)	1.83 (1.21–2.76)	2.75 (0.59–12.85)	0.197
Others	3 (1.0)	9 (1.8)	0.60 (.16–2.27)	4.24 (0.95–18.91)	0.058
Average monthly income					
<2500 ETB	104 (34.0)	346 (69.9)	1	1	<0.001
≥2500 ETB	202 (66.0)	149 (30.1)	4.51 (3.32–6.12)	2.87 (1.98–4.15)	
Knowledge					
Low	34 (11.1)	187 (37.8)	1	1	<0.001
High	272 (88.9)	308 (62.2)	4.86 (3.25–7.25)	3.12 (1.94–5.01)	

1, reference category; COR, crude odds ratio; AOR, Adjusted odds ratio; ANC, antenatal care; ETB, Ethiopian Birr.

## Discussion

This study assessed the extent of male involvement in the ANC utilization of their female partners and factors associated with their involvement in ANC utilization in the Bench Sheko zone, Ethiopia. The prevalence of male involvement in ANC was 38.2%. The findings are low compared to studies conducted in Addis Ababa (14), Arba Minch (17), and Myanmar (18). A possible explanation for this discrepancy might be that our study area included rural areas, whereas these studies were conducted in urban setting. Male partners who live in urban areas may be more likely to be involved in their spouses’ ANC utilization compared to those living in rural areas. However, our findings were higher compared to studies conducted in Anomabo, Central Region of Ghana (19), and Mekelle, Ethiopia (20), and the variation might be explained due to the different socioeconomic statuses of the regions.

Men who lived in urban areas had five times greater odds of being involved in the ANC utilization of their female partners. This might be due to individuals who live in urban areas being exposed to media and being close to health facilities. This result was in line with a study done in North Gondar that found urban residence to be positively associated with male involvement in maternal healthcare (11). Men aged 25–34 had two times greater odds of being involved in ANC utilization compared to younger men (15–24 years). This result was in line with a study conducted in northern Nigeria that found that young paternal age was a predictor of low male partner participation in maternity care (21). This result might be due to older partners having previous experience being involved in their female partner's ANC follow-up. This has programming implications. Young male partners may need extra support to be active participants in ANC. This finding suggests that new programs that target younger partners should be established in Ethiopia.

In addition, men with higher educational status and a monthly income of ≥2,500 ETB had four and three times the odds of male involvement in ANC utilization, respectively. This result was supported by studies conducted in Lemmo woreda of southern Ethiopia (22), North Gondar zone (11), Nepal (23), and Kenya (24) that found that those partners with high educational achievement and higher income showed more ANC involvement. Respondents who had high knowledge about ANC utilization had three times greater odds of involvement in the ANC utilization of their female partners. This result was in line with the study conducted in Arba Minch, Ethiopia (14), and Gulu District in Uganda (25), which found that partners’ knowledge about the advantage of ANC utilization showed a positive association with their involvement in their female partners’ ANC utilization.

This study has some limitations. The study used a cross-sectional design, and thus it is not possible to make causal inferences based on the data. Despite the fact, we tried to convince the respondents that the questions were used only for the study purpose; self-reported measures might have introduced social desirability bias. Despite this inherent limitation, the study provides useful information on male partners’ involvement on antenatal care services utilization that will inform health service planners to design strategies to improve maternal health in Ethiopia.

## Conclusion

Male involvement in ANC utilization in the study area is low. The type of residence (urban), age (25–34), education (diploma and above), income (≥2,500ETB), and high knowledge of the advantages of ANC were shown to be independently associated with male involvement in ANC utilization. Considering the importance of male involvement in MCH, we recommend advocating for policies and strategies that can improve the knowledge of men on the advantages of ANC and enhance their engagement in maternal care. In addition, interventions to increase male involvement should be carefully designed and implemented to mitigate the potential harmful effects on couple relationship dynamics (4). Special emphasis should be given to rural residents and those individuals with low income. Because the majority of the population in Ethiopia live in rural areas and have low access to media, policymakers should target younger populations in order to maximize male involvement in women's ANC utilization.

## Data Availability

Data will be available upon reasonable request from the corresponding author.
